# A Case of an Anomalous Tract Involving the Cecum After Open Right Inguinal Hernia Repair

**DOI:** 10.7759/cureus.22275

**Published:** 2022-02-16

**Authors:** Jessica Broderick, Thomas Siegel

**Affiliations:** 1 General Surgery, Beaumont Health, Dearborn, USA

**Keywords:** hernia repair complications, fistula to cecum, mesh migration, inguinal hernia repair, colocutaneous fistula

## Abstract

Inguinal hernia repair is a commonly performed surgical procedure and generally is well tolerated with minimal complications. We present the case of a 70-year-old male with an anomalous tract involving the subcutaneous tissue and cecum after an open right inguinal hernia repair via plug and patch approach. A partial cecectomy with appendectomy with excision of the tract was performed. While most complications are relatively minor and fistulas are quite rare, mesh migration is a possibility that should be considered during preoperative planning for recurrent hernia surgery.

## Introduction

Inguinal hernia repair is one of the most commonly performed surgeries and is typically well tolerated and effective, with minimal serious complications [1-3]. They have a 10%-15% recurrence rate with primary tissue repair, with a higher rate of recurrence for each subsequent repair [1]. The use of mesh has been studied extensively with a recurrence rate around 1%-2% [1]. Patients with mesh repair have also been found to have less postsurgical pain and an earlier return to work [2,4].

Two types of operations are frequently used today, plug and patch and tensionless mesh repair. In 1974, Lichtenstein and Shore first proposed the use of a mesh plug but later introduced the concept of tensionless repair with mesh to cover the defect without a plug or primary repair [1,5]. In 1993, Rutkow and Robbins proposed the plug and patch technique [1,6]. Originally, the plug was introduced without any securing sutures, but after cases of mesh migration, several securing sutures to the defect of the hernia or internal ring were recommended [5,6]. Presently, the operative technique is determined by surgeon preference, as they have similar rates of complications and recurrence [7].

Relatively minor complications typically arise from open inguinal hernia repair, such as seromas/hematomas (3.2%), urinary retention (10.6%), chronic pain (1.9%-12%), mesh infection (0.29%), and ischemic orchitis (0.2%-1.1%) [1,2,6,8-10]. Rarely do more serious complications develop, such as recurrence (1.89%) or mesh migration (approximately 3%) [8]. The migration of mesh into the abdominal cavity can result in abscess, fistula, or obstruction [3]. While infrequent mesh migration can cause profound long- and short-term sequelae, the risk should be acknowledged when determining the appropriate repair technique [3,11].

Complications due to mesh migration are much more likely to involve the sigmoid or small bowel than the cecum; cecal involvement is very rare [3,5]. An extensive literature review revealed only one case report on a cecal fistula after open inguinal hernia repair [5]. Here, we report a case of anomalous tract between the right inguinal subcutaneous tissues and cecum after an open right inguinal hernia repair with plug and patch technique, due to migration of mesh into the cecum.

## Case presentation

A 70-year-old male presented to the emergency department complaining of right groin swelling and pain after strenuous exercise at a stroke rehabilitation facility. Past medical history included cerebrovascular accident, remote myocardial infarction with stent placement, hypertension, and diabetes mellitus with neuropathy. He was a heavy tobacco smoker but denied alcohol or drug use. He also had a record of noncompliance with medical treatment. Ten years prior to this admission, he had bilateral open hernia repairs with mesh performed at an outside facility; no operative report was able to be obtained. Six years prior to this admission, he had a repair of a recurrent left indirect inguinal hernia with our hospital, which required a plug and two pieces of mesh to repair the large defect.

Physical exam revealed a mass with overlying skin erythema in the right inguinal region over the incision from the prior hernia thought to be a recurrent inguinal hernia. Laboratory tests showed mild anemia with hemoglobin of 12.0 but no other abnormal results. No imaging studies were performed at the time.

**Figure 1:**
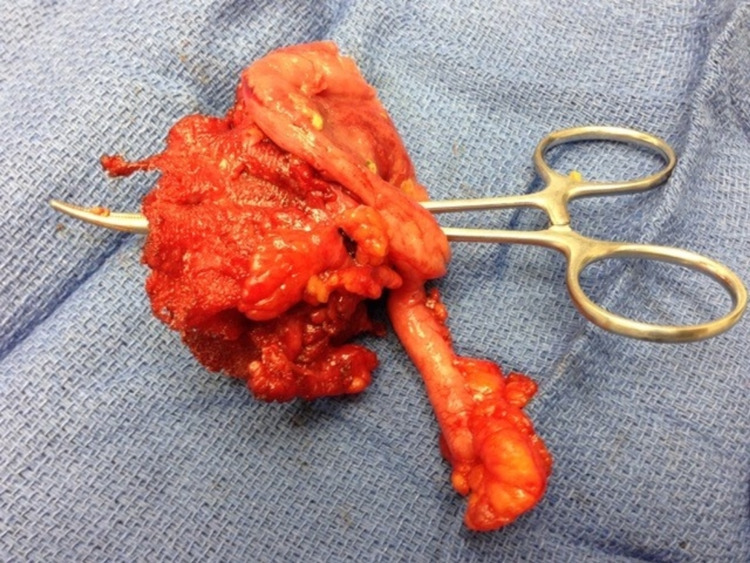
The existing plug and patch mesh was explanted en bloc with part of the cecum and the appendix after the mesh migrated into the cecum. The clamp is seen through the tract.

**Figure 2:**
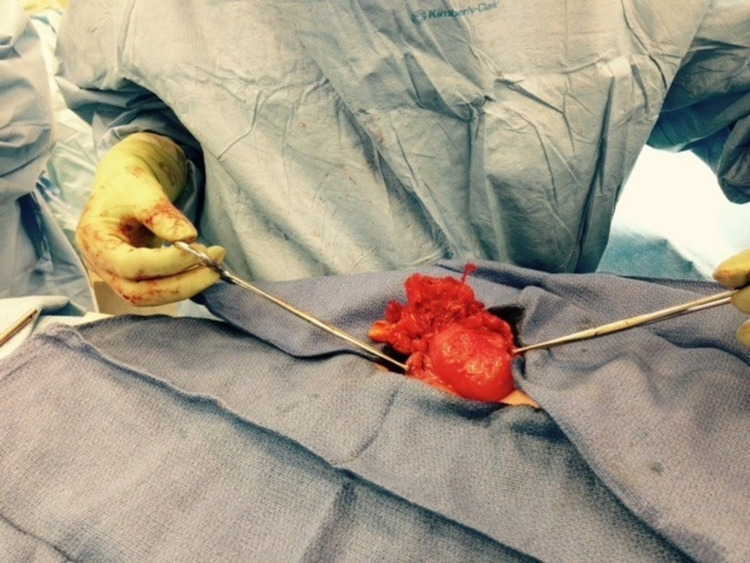
The existing plug and patch mesh was explanted en bloc with part of the cecum and the appendix after the mesh migrated into the cecum. The clamp is seen through the tract.

He was taken to the operating room for a right inguinal hernia repair. Upon exploration of the inguinal space, a hernia was not immediately identified; however, the mesh was foul-smelling and appeared to be contiguous with bowel perforation. The densely adherent mesh was dissected free from surrounding tissues and was found to be connected at the most distal part of the cecum, including the appendix but not involving the terminal ileum, as well as the epithelial surface of the skin. The old plug and patch mesh was explanted en bloc with part of the cecum and the appendix, pictured below in Figures 1, 2. The mesh had migrated into the cecum and formed a tract from cecum to the right inguinal region subcutaneous tissue. The entire procedure was undertaken transinguinally. The fascial defect was closed primarily, in the manner of Cooper, joining the conjoint tendon to the iliopectineal line with a relaxing incision. The skin and subcutaneous tissues were closed with interrupted nylon vertical mattress sutures and packed with iodoform gauze. Wound cultures grew heavy *Escherichia coli*, *Proteus *species, and *Streptococci *species.

Postoperatively, his course was complicated by a prolonged ileus, urinary retention, and a chronically infected right inguinal wound. At the time, computed tomography angiography (CTA) performed for bilateral lower extremity weakness and pain showed chronic occlusion of the abdominal aorta with collateralization, and an axillobifemoral bypass was planned for when the groin incision healed. Over the next two years, multiple attempts were made to encourage healing, including packing, wound vac, and wet-to-dry dressings. When seen in office after two years, the wound had finally healed. A year later, he presented for an office visit with a small area of granulation tissue at the medial aspect of the right inguinal incision, which was managed with silver nitrate cautery. An ultrasound showed no evidence of fluid collection. Physical exam revealed an intact repair with no signs of recurrence. He has been seen recently, at which time the wound was fully healed and the repair remains solid.

## Discussion

A case of an anomalous tract involving the cecum and subcutaneous tissue after an open right inguinal hernia repair using the plug and patch technique was presented. Upon review of the literature, we have found seven cases of colocutaneous fistulas after open inguinal hernia repairs, presented below in Table 1. Six of these involved a left inguinal hernia and the sigmoid colon; five of the six utilized the plug and patch technique [1-3,6,11] and the remaining one employed the Lichtenstein technique [12]. Only one of the seven cases involved the cecum with plug and patch of a right inguinal hernia repair [5]. The fistulas presented at a mean of five years after surgery and a median of three years. The longest was recognized 13 years after repair [5], and the shortest at two years [2,6]. Our patient’s tract was found at 10 years postoperatively.

**Table 1:** Cases of colocutaneous fistulas.

Case	Years postoperatively	Laterality	Technique	Involved segment
1 [1]	3	Left	Plug and patch	Sigmoid
2 [2]	2	Left	Plug and patch	Sigmoid
3 [3]	9	Left, sliding	Plug and patch	Sigmoid
4 [12]	3	Left	Lichtenstein	Sigmoid
5 [11]	3	Left	Plug and patch	Sigmoid
6 [6]	2	Left	Plug and patch	Sigmoid
7 [5]	13	Right	Plug and patch	Cecum

A case of an incisional hernia with fistula to cecum [8], three cases of mesh migration to sigmoid with no evidence of fistula [4,13,14], a case of mesh migration to cecum with subsequent formation of colovesical fistula [15], and a case of mesh migration to the cecum with no evidence of remaining tract [16] were of interest but excluded from data points. Cecal involvement appears to be very rare, likely due to a few anatomical reasons. The cecum is more protected and distant from the internal ring than the sigmoid; therefore, it is in less contact with the mesh [5]. It is also more firmly fixated to the retroperitoneum [5].

Primary mechanical migration is due to inadequately secured mesh, whereas secondary is due to chronic inflammation [8]. There are many plausible risk factors for mesh migration and/or fistula formation. These can be mechanical in nature, including inadequate initial anchoring, unsatisfactory placement of the mesh plug, direct contact between mesh and bowel, and type of hernia [1-6,8,11,12].

Inadequate initial anchoring could be due to poor fixation without sutures, with absorbable sutures, or suturing to low-quality tissue [1-5]. There is wide agreement throughout the reviewed studies that the mesh plug should be sutured either to the defect or the internal ring [1,4]. If the suture used is absorbable, it will increase the risk of migration [1,4]. With the inherent weakness of the transversalis fascia and altered collagen formation, poor surrounding tissue is likely and has to be taken into account when suturing the plug in place [4]. The stability of the repair during the first couple weeks in the postoperative period is important to overall stability, and the initial inflammatory reaction is essential to fibrose plug into place [1]. If the patient is unable to mount an appropriate inflammatory response, enough fixation may not occur to ensure the mesh does not migrate [1]. The mesh cone should induce an inflammatory response and cause the tissues to fibrose around it [4]. Different types of mesh have been studied, with a much larger inflammation reaction occurring with polypropylene vs polytetrafluoroethylene mesh [1,12,17], resulting in a higher degree of fibrosis. The amount and character/condition of inflammation was proportional to the ratio of foreign material (polypropylene) in the mesh and to the surface area in contact with the mesh [17]. Polypropylene is used quite frequently as it is relatively cheap and easy to find, as well as creates a significant tissue reaction for fibrosis [8].

Unsatisfactory placement of the mesh plug can occur. The profile of the cone may be too long and protrude into the peritoneal cavity or be placed too deep into the defect with a resultant increase in pressure on the peritoneum [3]. If the hernia sac is simply inverted, it may also be stretched and compromise the tissue [6]. If the tissue is weak or not intact, there could be direct contact of mesh and bowel, which can lead to pressure necrosis and eventual erosion into the bowel [3,8]. Avoiding damage to the peritoneal sac and therefore avoiding direct contact of bowel and mesh is essential [5]. A heavyweight mesh may be more likely to cause the erosion [3]. In sliding hernias, a retroperitoneal organ forms part of the wall of the hernia sac, increasing contact surface area and placing them at an inherently higher risk of fistula [3,12].

Comorbidities may also increase the risk of fistula due to increased inflammation, poor wound healing, or immunosuppression (diverticulitis, diabetes, obesity, vascular disease, tobacco abuse, chemotherapy, or steroid use) [1-6,8,11,12]. The patient in our case study did have poorly controlled diabetes as well as extensive vascular disease discovered postoperatively.

Mesh infections can contribute to fistula formation as well [12]. Chronic infection rates are affected by the type of mesh, surgical technique chosen, and antibiotic prophylaxis [12]. Infections can lead to chronically draining sinus tracts, abscesses, adhesions, and/or migration of the mesh leading to fistula formation [12].

There is some contention over which technique is optimal, plug and patch vs Lichtenstein tensionless repair. Of the seven case reports with fistulas, only one was after a Lichtenstein repair [12]. While earlier case studies suggested a plug may not be necessary and the mesh alone was safe and equally effective for small to medium inguinal hernias [18], later studies report conflicting data [7,19]. One case found equivalent complications and outcomes between the two techniques [19]. Another study showed the techniques to have similar rates of complications/recurrence, but the plug and patch repair patients had less postoperative pain, a better quality of life, shorter operative time, and a shorter hospital stay [7]. Currently, either repair is equally effective and could be used for inguinal hernia repair [7]. The possibility of avoiding a plug altogether, using a bioabsorbable plug, using a connected prosthesis, or reshaping the mesh cone to eliminate the sharp point could be addressed in future studies [2,3,11]. If the plug migrating is a major concern due to high-risk patient characteristics, one could consider doing a Lichtenstein or primary repair of those at high risk of fistula formation [1]. The risk of recurrence with a primary repair would need to be considered and weighed against the risk of fistula formation. At a 10%-15% recurrence rate, it would only be sensible in those at an extremely elevated risk for fistula. Many discuss primary repair preoperatively, but in most patients, it still is going to be more beneficial to use mesh, with either the Lichtenstein or plug and patch repair [1].

These patients can present with nonspecific symptoms such as abdominal pain or nausea/emesis as well as more concerning findings of overlying skin changes, abscesses, perforation, fistulas, or obstruction [12]. If mesh migration is suspected, it is best detected with colonoscopy [12]. We need to have a high index of suspicion in those with prior hernia repairs with possible recurrences, especially in higher-risk individuals as outlined above [1].

## Conclusions

In conclusion, we have presented a case of an anomalous tract between the right inguinal subcutaneous tissue and cecum after inguinal hernia repair. Cecal involvement appears to be very rare, with only one case report of a colocutaneous fistula involving the cecum. Cases of mesh migration may be due to patient comorbidities, tissue quality, mesh infection, or mechanical in nature. A greater index of suspicion is needed in high-risk patients with prior hernia repairs when presenting with nonspecific abdominal symptoms. While most complications are relatively minor and fistulas are quite rare, complications due to mesh migration are a possibility that should be acknowledged during preoperative planning.
